# Global Monitoring of Water Supply and Sanitation: History, Methods and Future Challenges

**DOI:** 10.3390/ijerph110808137

**Published:** 2014-08-11

**Authors:** Jamie Bartram, Clarissa Brocklehurst, Michael B. Fisher, Rolf Luyendijk, Rifat Hossain, Tessa Wardlaw, Bruce Gordon

**Affiliations:** 1 Department of Environmental Sciences and Engineering, University of North Carolina, Chapel Hill, NC 27599, USA; E-Mails: clarissa.brocklehurst@gmail.com (C.B.); fishermb@email.unc.edu (M.B.F.); 2 The United Nations Children’s Fund (UNICEF), New York, NY 10017, USA; E-Mails: rluyendijk@unicef.org (R.L.); twardlaw@unicef.org (T.W.); 3 Division of Public Health and the Environment, World Health Organization, Geneva 1211, Switzerland; E-Mails: hossainr@who.int (R.H.); gordonb@who.int (B.G.)

**Keywords:** water, sanitation, monitoring, JMP, international, method, WHO, UNICEF, Millennium, MDG

## Abstract

International monitoring of drinking water and sanitation shapes awareness of countries’ needs and informs policy, implementation and research efforts to extend and improve services. The Millennium Development Goals established global targets for drinking water and sanitation access; progress towards these targets, facilitated by international monitoring, has contributed to reducing the global disease burden and increasing quality of life. The experiences of the MDG period generated important lessons about the strengths and limitations of current approaches to defining and monitoring access to drinking water and sanitation. The methods by which the Joint Monitoring Programme (JMP) of WHO and UNICEF tracks access and progress are based on analysis of data from household surveys and linear regression modelling of these results over time. These methods provide nationally-representative and internationally-comparable insights into the drinking water and sanitation facilities used by populations worldwide, but also have substantial limitations: current methods do not address water quality, equity of access, or extra-household services. Improved statistical methods are needed to better model temporal trends. This article describes and critically reviews JMP methods in detail for the first time. It also explores the impact of, and future directions for, international monitoring of drinking water and sanitation.

## 1. Introduction

International monitoring of drinking water and sanitation is carried out in response to international development policies, while also generating knowledge that both informs the development and facilitates the implementation of those same policies [1,2]. Monitoring programmes track global, regional, and national progress on expanding access to drinking water and sanitation, and highlight gaps and opportunities for accelerating that progress.

International monitoring of drinking water and sanitation has been on-going since the 1930s, when such monitoring was carried out by the League of Nations Health Organization; subsequently by the World Health Organization (WHO), and now jointly by WHO and The United Nations Children’s Fund (UNICEF) through their Joint Monitoring Programme (JMP). In recent decades, this monitoring has been conducted in support of global targets established under the UN system through the second United Nations’ (UN) Development Decade (the 1970s); the International Drinking-water Supply and Sanitation Decade (the 1980s); the World Summit for Children [3]; and the Millennium Development Goals (established in 2000 for the period 1990–2015) [4]; as well as the International Decade for Action: Water for Life (2005–2015) [5] and the International Year for Sanitation (2008, Figure 1) [6]. 

**Figure 1:**
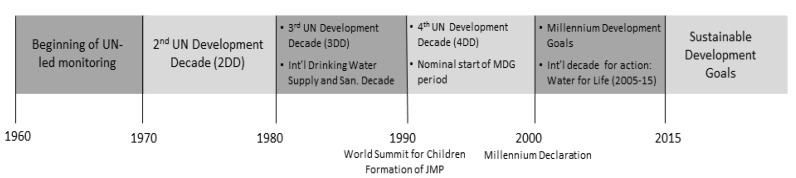
Timeline of international targets and actions related to drinking water and sanitation.

Since monitoring under the UN system began, the global water and sanitation landscape has changed dramatically as a result of these policies and their underpinning national initiatives, as well as of major demographic and technological shifts. Since 1970, the global population has nearly doubled, while the urban population almost tripled [7]. The number of people using improved sources of drinking water expanded from 2.4 billion in 1970 to 6.2 billion in 2012 (64% to 89% coverage), while the number using basic sanitation increased from 1.3 to 4.4 billion (36% to 64%). More than half of the world’s population now gets water from a piped source in the home [8]. Life expectancy at birth has increased from 56.5 to 68.7 years [7], due in part to improvements in drinking water and sanitation [9,10]. 

After nearly 80 years of monitoring programs, it is appropriate to reflect critically on the methods by which this monitoring has been conducted; the evolution of these methods over time; and to consider the changes and improvements in monitoring methods needed to respond to the world’s future drinking water and sanitation situation. These needs are very different from those that the existing system evolved to address [11]. This paper documents the evolution of international drinking water and sanitation monitoring, describing in detail for the first time the method currently applied, analysing the strengths and limitations of the current approach, as well as potential alternatives, and reflecting upon potential steps to ensure its future relevance. A more detailed exploration of the policy implications of current and proposed future monitoring approaches is presented in a forthcoming companion article [12].

## 2. Background

### 2.1. History of International Drinking Water and Sanitation Monitoring

As early as 1930, the League of Nations Health Organization (LNHO, predecessor of the WHO), published recommendations and collected data on drinking water and sanitation under its rural hygiene programme. These recommendations coincided with the LNHO’s shift in focus during the 1930s to emphasise “social medicine” (*i.e.*, public health), and were supported by formal and informal monitoring activities, focusing largely on Europe and Asia [13]. United Nations (UN)-led monitoring of drinking water and sanitation began in the 1960s [14,15], compiling early reports from annual questionnaires sent by WHO to the Ministries of Health of monitored countries. These reports documented coverage of households with drinking water and sanitation technologies, using categories similar to those used today (Table 1), and also described the water and sanitation sectors in the countries concerned. 

**Table 1:** JMP categorisation of drinking water and sanitation coverage according to use of different facility classes. Also shown are core questions on water, sanitation, and hygiene.

**(1a) Drinking Water**		
Drinking water core questions (1)		
What is the main source of drinking water for members of your household? Where is that water source located? How long does it take to go there, get water, and come back? Who usually goes to this source to collect the water for your household? Do you do anything to the water to make it safer to drink? (Introduced 2005) What do you usually do to make the water safer to drink? (Introduced 2005)		
**MDG Categorisation of Households (2)**	**JMP Disaggregated Categorisation of Households**	**Underlying Questionnaire Responses**
Not using an improved drinking water source	Collection of water from a surface water source	Surface water (river, dam, lake, pond, stream, canal, irrigation channel)
	“Other unimproved sources”	Unprotected dug well Unprotected spring Cart with small tank or drum Tanker truck (3) Bottled water where other water source is classified as unimproved (4)
	Piped drinking water into dwelling, plot or yard	Piped water into dwelling, yard or plot
**(1b) Sanitation**		
Sanitation core questions (1)		
What kind of toilet facility do members of your household usually use? Do you share this facility with others who are not members of your household? With how many households do you share this facility? The last time (Name of Child U5) passed stools, what was done to dispose of the stools?		
**MDG Categorisation of Households (2)**	**JMP Disaggregated Categorisation of Households**	**Underlying Questionnaire Responses**
Not using improved sanitation	open defecation	No facilities, bush or field, open water bodies(open defecation)
	Unimproved	Flush or pour-flush to elsewhere (that is, not to piped sewer system, septic tank or pit latrine) Pit latrine without slab, or open pit Bucket Hanging toilet or hanging latrine
	Shared use of a facility otherwise classified as ‘improved’	Use of facilities listed below where shared by more than one household
Using improved sanitation	Improved sanitation	Flush or pour-flush to piped sewer or septic tank or latrine pit Ventilated improved pit (VIP) latrine Pit latrine with slab Composting toilet
**(1c) Hygiene**		
Hygiene core question (5)		
Can you please show me where members of your household most often wash their hands? (Observe presence of soap, water) Do you have any soap or detergent (**or other locally used cleansing agent**) in your household for washing hands? (MICS only)		
**MDG Categorisation of Households (2)**	**JMP Disaggregated Categorisation of Households**	**Underlying Questionnaire Responses**
Using adequate hygiene	Adequate hygiene supplies	Presence of soap and water for handwashing
Not using adequate hygiene	Inadequate hygiene supplies	Absence of soap, water, or both in handwashing process

Notes: (1) Core questions on drinking water and sanitation. These have been used with few changes since the introduction of the MICS and DHS surveys, except as noted. [16]. All questions are self-report. (2) The terminology here is that of the MDG indicators. The wording of the target in contrast refers to “sustainable access to safe drinking water and basic sanitation” [17]. (3) Water provided by Tanker Truck is considered adequate in the United Arab Emirates, Oman and Kuwait as it is a regulated service by the drinking water authorities delivering water to nomadic populations and communities not connected to a piped network. (4) Bottled/packaged water is considered ‘improved’ only when the household uses drinking water from an improved source for cooking and personal hygiene. Where information on the other source is not available the household is categorised as using piped water. For countries with information about the secondary source, more than 80% of “bottled water users” report having water piped on premises (5) MICS [18]. First question also used in DHS [19].

Information provided by national authorities was often incomplete and usually did not elucidate definitions of access. Furthermore, estimates varied considerably between reporting years, and independent verification of information was rarely possible.

In 1990, WHO and UNICEF combined monitoring efforts into a “Joint Monitoring Programme for Water Supply and Sanitation” [20]. While referred to as a joint programme, the JMP has functioned since its inception through coordination of activities carried out separately by the headquarters of the two organizations.

In 1997, WHO and UNICEF reviewed their monitoring experience and agreed on a future strategy. An important change was made to the approach for estimating coverage, implemented for the Year 2000 JMP Report; switching from government-provided data to data collected through censuses and nationally-representative household surveys. While censuses were well-established, the late 1990s had seen increasing availability of household survey data, largely due to the implementation of Multiple Indicator Cluster Surveys (MICS) by UNICEF and the Demographic and Health Surveys (DHS) by the United States’ Agency for International Development (USAID). The shift to household surveys and censuses was expected to be low-cost (because primary data collection was undertaken by others) and likely to contribute to more accurate coverage estimates (because data were from users of facilities). The 2000 JMP report contained both country sector review information based on government-provided data and assessments of coverage with drinking water and sanitation based on household survey data [21] wherever available. The collection of information from national authorities was subsequently abandoned and, as a result, subsequent JMP reports focused on coverage, and have not reported on other aspects of the sectors. 

### 2.2. International Targets and Agreements on Drinking Water and Sanitation

Since the beginning of international drinking water and sanitation monitoring in the 1930s, monitoring has been carried out in response to, and in support of a series of international targets and agreements around drinking water and sanitation (Figure 1). The International Drinking-water Supply and Sanitation Decade (1981–1990), which had as its declared objective “substantial improvement in drinking water and sanitation by 1990” [22], and resulted in national action plans for drinking water supply and sanitation, as well as the Mar del Plata action plan. This decade also led to increased emphasis on the participation of communities in the management of water and sanitation facilities [23]. The UN tasked WHO to report on progress in extending access to drinking water and sanitation during the Decade [22], and WHO responded by issuing reports based on drinking water and sanitation coverage statistics provided by national authorities, as well as on key sector attributes.

In 1990, the World Summit for Children, with its associated Declaration and Plan of Action [3,24], called for universal access to safe drinking water and sanitary means of excreta disposal by 2000. The Summit resulted in ratification by 192 countries of the Convention on the Rights of the Child. The demand for nationally representative information resulting from this process spurred UNICEF to increase its monitoring efforts, initiating MICS in 1995 to assess the situation of countries with respect to diverse concerns, including drinking water and sanitation [25]. 

In September 2000, world leaders adopted the Millennium Declaration [4]. The associated Millennium Development Goals (MDGs), first presented in 2001, set out time-bound targets for several components of development policy [26]. The target concerning drinking water and sanitation was repeatedly edited until adopted in its final form in 2006 as Target 7C: to halve, between 1990 and 2015, “*the proportion of the population without sustainable access to safe drinking water and basic sanitation*” [6,17,27]. Wording changes were substantive and are germane to this paper. Notably:

- The original wording addressed only drinking water; sanitation was added to the target after the 2002 World Summit for Sustainable Development [28].
- Reference to affordability was included in the original (Millennium Declaration) wording [4], was repeatedly deleted and re-inserted, and was eliminated in the final version [4,6,17,27,28,29,30].
- Reference to “safe” drinking water was sequentially added and removed and was retained in the final version [5,6,17,27,29,30].
- Reference to “sustainable access” was edited in and out and eventually retained.
- The formulation of “halving the proportion of the un-served” was consistently used.

The JMP, as the only available source of comprehensive and internationally-comparable information on drinking water and sanitation coverage, served as the UN-recognised instrument for monitoring progress towards the MDG target [20]. Indicators for MDG monitoring were agreed in 2006 [17] based on recommendations from WHO and UNICEF in light of JMP approaches. 

While human rights to basic services were referred to in the 1992 Dublin Statement on Water and Sustainable Development [31], and subsequently in the Millennium Declaration [4], the human right to drinking water and sanitation was not mentioned in the text of the MDGs [17]. However, the human right to water was recognised, initially in 2002 through General Comment 15 of the UN Committee on Economic, Social and Cultural Rights [32], and the human right to both water and sanitation was subsequently recognised in 2010 through UN General Assembly and UN Human Rights Council resolutions [33,34]. This recognition clarified the parameters by which adequacy of drinking water and sanitation was to be judged, and increased demand for analytical approaches capable of measuring equality and identifying discrimination. JMP responded with wealth quintile analysis in its 2004, 2010 and 2012 reports [35,36,37], on-going analysis of urban-rural disparities; and efforts to understand the rate of progress achievable in response to the concept of “progressive realisation” [38].

### 2.3. Use of International Drinking Water and Sanitation Monitoring Data

As the MDGs—and drinking water and sanitation within them—gained momentum in the 2000s, greater attention was applied to policies and financing. Regional policy initiatives emerged, especially on sanitation [39,40,41,42]. These created demand for the kind of information on policies, constraints, and resource flows previously collected by WHO and JMP from national authorities. 

In the mid-2000s, “UN-Water” was established as the first of a new model of UN system coordination arrangements [43,44]. One of its earliest decisions was to recognise the JMP reports as being prepared by WHO and UNICEF on behalf of the associated UN entities (of which there are now 27).

A call to action by the UK’s Department for International Development (DfID) [45] led to two linked initiatives: the “Global Annual Assessment” of sanitation and drinking water (“GLAAS”, now renamed the Global Assessment and Analysis of Sanitation and Water) [46] and the Sanitation and Water for All partnership [47]. 

GLAAS is implemented by WHO under the aegis of UN-Water to analyse progress and obstacles to that progress in the sector (a task performed by JMP prior to the switch to household data), using existing high-quality sources of consolidated data, such as OECD for aid flows [48], supplemented by questionnaire surveys of national authorities for other data.

The Sanitation and Water for All (SWA) partnership of governments, NGOs, and other stakeholders depends on data from JMP and GLAAS to provide the evidence and accountability needed to improve drinking water and sanitation in developing countries. Other initiatives based on mutual accountability, most notably the regional sanitation conferences [39,41,42,49,50], also rely heavily on JMP data. 

Data from JMP are also used in reports by the UN Secretary General, the United Nations Development Programme (UNDP) and the World Bank, as well as by WHO and UNICEF. JMP data are also used in composite indices and comprehensive reviews such as UNDP’s Human Development Report [51], including its water theme review [52]; the World Water Assessment Programme [53]; the Ibrahim Index of African Governance [54]; and UN Habitat’s slum population analyses [55]. JMP data have been used in estimating the global burden of disease associated with water and sanitation [10,56,57,58] and to assess the cost-effectiveness, benefit-cost ratio, global expenditure, and investment needs in global drinking water and sanitation [57,59,60].

While JMP data have been widely used, JMP’s methods have also been repeatedly criticised [61,62,63]. These criticisms are explored in Section 6 below, alongside other methodological limitations that have not been previously discussed in print. 

## 3. Methods

From the 1960s until 2000, data collection was through distribution of questionnaires to national authorities. In some cases, one person would complete and return the questionnaire, in others, ministries and departments would cooperate to compile responses; in some cases, support was provided by UN agencies and in others not. Quality control comprised review of completed questionnaires for internal consistency and consistency with previous returns, often leading to iteration between the WHO officer overseeing the programme and national counterparts. As summarised above, the 2000 report marked a substantive change in the JMP method, which has evolved subsequently. 

### 3.1. Sampling and Data Collection Methods 

The JMP now compiles and analyses data on all UN Member States, and UN-recognised countries and territories for which data are available (over 190 of the 240 UN-recognised countries and territories, as of 2014). The principal data sources used by JMP are national censuses and nationally representative household surveys. 

National censuses are undertaken by many countries at ten-year intervals. They typically collect information on the country’s entire population, through questionnaires and/or interviews. They normally include far fewer questions in total than household surveys and may or may not address drinking water and sanitation.

Nationally-representative household surveys are undertaken periodically in over 100 countries (Table 2). They are typically conducted by national statistics offices, often with support from foreign or international agencies (Table 2). The decision to undertake a DHS or MICS survey is made by national governments in consultation with USAID or UNICEF, and some countries use modules from each; LSMS surveys are administered by national governments in partnership with the World Bank according to the data requirements of both parties. These nationally representative household surveys include extensive quality assurance and quality control procedures at the field-level to ensure data validity [64,65,66,67]. For surveys such as MICS and DHS, a (usually two-level) stratified randomised cluster approach is used to select households for interview. Briefly, each country is divided into several hundred Primary Sampling Units (PSUs) of approximately equal population, based on data from the most recent national census, where available. Within each PSU, households are enumerated and randomised; several (typically 10–35) randomly selected households in each PSU are identified to be surveyed. Samples are stratified by important geographic (e.g., urban/rural) and occasionally demographic variables to increase homogeneity, and thereby minimise sampling variance [68,69]. In selected households, the head of household or another resident adult is asked to engage in an interview lasting 45 min to 1.5 h, in which questions similar to those in Table 1 are asked, among others. Like national censuses, household surveys have a limited set of questions on drinking water and sanitation: ten of almost 400 questions in MICS questionnaires and ten of the 850 questions that constitute all DHS question modules focus on these themes, and the LSMS has a similarly limited set of drinking water and sanitation questions. The most recent round of MICS surveys had an average cost of USD 750,000 per country, while DHS surveys typically cost USD 2–2.5 million. The cost of a national census is dependent on the size and density of the population surveyed. 

**Table 2:** Nationally representative household surveys and other data sources that include data related to drinking water and/or sanitation and are used by JMP.

Survey or Data Source	Supported By (1)	Initiated (Year)	Total Number of Surveys in JMP Database (to End–2012)	Scale	Source Reference for Method Description
Demographic and Health Surveys (DHS)	USAID	1985	259	Conducted in 7000—30,000 households in each of 85–90 countries, typically at 5 year intervals, more frequently in some countries	[70]
Multiple Indicator Cluster Surveys (MICS)	UNICEF	1995	172	Conducted in 5000—15,000 households in each of 85–90 countries initially at 5-year intervals now at 3-year intervals	[64]
World Health Surveys (WHS)	World Health Organization	One round in 2003; Beginning in 2010, WHO initiated Study on global Ageing and adult health (SAGE, [71]) considered to be the second round of WHS	45 surveys in the developing countries with WASH data	Conducted in 5000–15,000 households	[72]
Living Standards Measurement Study (LSMS)	World Bank	1985	80	Approximately 5 surveys per year across 36 developing countries	[73]
National censuses	Variable	n/a	252	Every 5–10 years; most censuses target all households	[74]
Other household surveys	Variable	n/a	655	Variable	Following similar methodology as DHS and MICS above
Developed country coverage reports	National authorities, often the line ministries, validated by national statistical offices	n/a	334	Usually conducted yearly	n/a

Note: (1) “support” normally entails both financing and technical advice.

The JMP prefers to base its monitoring on analysis of “raw” household-level data. However, in approximately half of surveys and censuses, such data are unavailable and “survey reports” or “census reports” prepared by national statistical offices or other survey authorities are used. These survey and census reports contain data tables and short narratives presenting the main findings of the survey, which may include data comparisons with previous surveys and explanations of changes. 

When data from censuses and household surveys are not available, for example in some developed countries where censuses no longer collect information on drinking water and sanitation, data reported by national government agencies are used, after review for internal consistency and for consistency with previous reports.

### 3.2. Categorisation of Households by Drinking Water and Sanitation Facilities Used

The JMP categorises households according to the types of drinking water source and sanitation facilities used (Table 1). While the categories and their definitions have evolved over time, this basic approach has underpinned monitoring since the 1960s, if not earlier. Drinking water sources and sanitation facilities are determined by respondents’ self-report, assisted by pictures of different facility types. Enumerators are also trained and tested using these pictures, and their work checked during piloting and data collection, to ensure accurate categorisation of facilities.

The task of measuring coverage by facility type is complicated by inconsistent use of drinking water and sanitation terminology between, and sometimes within data sources used by the JMP. In some censuses and surveys, especially earlier ones, response options for drinking water or sanitation facilities do not coincide with the facility classes used by JMP. For example, many have a response option “well” which includes both protected and unprotected wells. JMP corrects for this loss of information by interpolating the proportion of households using protected wells from another survey for the same country that contains disaggregated information by well type; usually the survey closest in time to the missing data. In countries for which no surveys provide such information, 50% of facilities are estimated to be improved. Such a correction was applied to one or more surveys in more than 100 countries and territories as of 2012 (Table 3). 

**Table 3:** Frequency of correction of surveys to account for differences between census/survey and JMP classes of water and sanitation since the start of JMP reporting.

Survey/Census Class	JMP Classes	Countries and Territories for Which One or More Surveys Have Been Adjusted
Well	Protected well Unprotected well	106
Spring	Protected spring Unprotected spring	80
Piped water	Piped into dwelling, plot or yard Public tap or standpipe	53
Traditional latrine Latrine Pit latrine Pit Simple pit Shallow pit	Pit latrine with slab Pit latrine without slab Ventilated pit latrine Open pit	112

In addition, some common terms are used inconsistently among different surveys. For example, the terms “open pit”, “pit”, and “traditional” latrine have each been used in various data sources to describe facilities of the same type, and the term “covered latrine” has been used variably to refer to latrines with a roof, a pit cover or a covered drop-hole. The problem is extensive: in a study for the World Bank, Günther and Fink [75] found “*over 400 different sanitation codes in the DHS surveys*” and that “*Water categories were even more heterogeneous, with over 500 different codes across 172 DHS surveys alone*”. Accuracy is lost when statistics from surveys and censuses using different classification systems are compared or aggregated unless each system can be mapped to a common set of definitions. The JMP consults with local survey managers and national sector experts to verify the meaning of different terms in their local context, map each to its corresponding classification, and treat data accordingly. 

Once these corrections have been made, data on up to ten variables are extracted from all censuses and surveys for which they are available. These are the proportions of rural and urban households that use: piped drinking water on premises; any drinking water facility classified as improved (including piped on premises); water collected from a surface water source; any sanitation facility classified as improved; and no sanitation facility (households considered to practice open defecation).

### 3.3. Statistical Analysis of Data to Estimate Status and Identify Trends 

**Figure 2:**
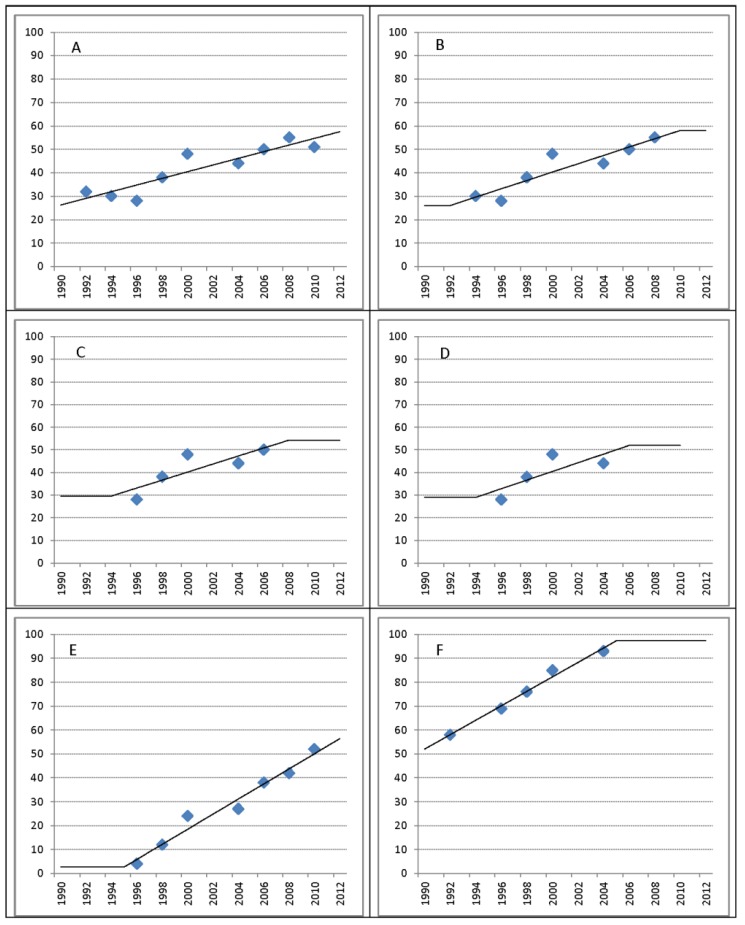
Illustration of JMP regression line extrapolations for countries with missing data. (**A**) 9 data points (1992–2010); extrapolation 2 years; 1990 estimate: 26.3%; 2012 estimate: 57.6%. (**B**) 9 data points (1994–2008); Extrapolation 2 years + 2 years flat; 1990 estimate: 26.1%; 2012 estimate: 58.1%. (**C**) 5 data points (1996–2006); Extrapolation 2 years + 4 years flat; 1990 estimate: 29.6%; 2012 estimate: 54.3%. (**D**) 4 data points (1996–2004); Extrapolation 2 years + 4 years flat; 1990 estimate: 29.0%; 2012 estimate: Not available. (**E**) Flat below 5% coverage; (**F**) Flat after 95% coverage.

For each country, an ordinary least squares linear regression is conducted for each of the ten proportions described above as a function of time (year of survey). Coverage rates are reported based on these regressions. Regression lines are extrapolated for up to two years before the earliest and after the most recent census or survey year, (but constrained within the coverage range 0%–100%). For coverage in the range 5%–95%, the coverage at the end of the two-year extrapolation is then reported for up to four further years, after which no further data are reported (*i.e.*, reports show “data unavailable”); for countries outside this range, the continuation is extended indefinitely (Figure 2). 

The rural and urban coverage rates reported by JMP are the proportions of households using the drinking water or sanitation facility classes described in Table 1. These values are estimated from the corresponding linear regression for the year of interest for: on-plot piped drinking water (piped water in the home, yard, or plot), any improved drinking water source (a category that includes on-plot piped), and water collected from a surface water source, while the proportions for unimproved sources are calculated as those not covered by any improved drinking water source.

The proportions of rural and urban households where open defecation is practiced are estimated using the corresponding regression lines in the same way. Data on the sharing of toilets between households are available in a minority of more recent censuses and surveys (for 128 developing countries, plus an additional 28 industrialised countries that report having no sharing of toilets among households as of 2013). The mean proportions of the rural and urban populations living in households that share toilets in each country are calculated from all surveys in which this information is collected. These values, expressed as percentages, are the coverage estimates for shared use of improved sanitation, and are applied across all time points. 

For MDG monitoring, households are categorised as using improved sanitation if they use a facility that is both classified as improved and is not shared with other households (Table 1). This is estimated by adjusting the estimates of use of any class of improved sanitation to account for proportions of households that share facilities. This adjustment was applied to 128 (of 193) countries and territories for which sanitation estimates are given in the 2013 report. For countries with no surveys providing information about the use of public or shared facilities (including 47 of the 52 developed countries), all households are categorised as not sharing toilets.

A similar adjustment is made for water safety for one country only: Bangladesh. The proportions of households using tubewells/boreholes and protected dug wells are reduced by the proportion of such wells that exceed the Bangladesh national standard for arsenic in drinking water of 0.05 mg/L, which is higher than the WHO provisional guideline value of 0.01 mg/L. An adjustment of 27%, based on the first nationwide arsenic survey [76], was first made in 2004. This proportion decreased to 20% [77] and most recently 13% [78]. Households so treated are considered to use an “unimproved” water facility for the purposes of MDG monitoring and an unimproved/non-surface source for disaggregated reporting (Table 1). 

For each country or territory, the populations using the drinking water and sanitation facility classes described in Table 1 are estimated separately for the rural and urban populations; national coverage rates are estimated by calculating a population-weighted average of urban and rural coverage using the most recent population estimates of the UN Population Division [79].

## 4. Reporting

The JMP produces biennial coverage reports that have become progressively more detailed since 2002. The 2014 report provides drinking water coverage estimates for 199 and sanitation coverage estimates for 193, countries and territories, representing 98.7% and 97.8% of the global population, respectively, and reflecting the two levels of aggregation described in Table 1 [8]. These reports now also include regional and global coverage estimates and progress assessments for countries with respect to the MDG target sub-components of drinking water and sanitation (“on-track/off-track” analysis).

*Regional estimates* are made by summing the rural and urban populations using the required drinking water and sanitation classes in all countries for which data are available for the region of analysis, and expressing this as a percentage of the total population of those countries. If those countries contain at least 50 percent of the regional population, these proportions are applied to the regional population and to corresponding sub-populations. These calculations are carried out for MDG regions [20], and for regions defined by WHO and UNICEF. Similar calculations have been carried out for Africa as defined by the African Union [80], and for Arab States [81]. 

*Global coverage* is estimated as the sum of the population-weighted MDG regional coverage estimates, divided by the global population. 

*Rural vs. Urban coverage* is estimated using national-level disaggregated data. Currently, no internationally consistent definition of the distinction between rural and urban settlements exists, with each country or region using its own definition, often based on the population of the settlement [82,83].

*Progress* towards the MDG target is assessed separately for drinking water and sanitation in each country. The national coverage estimates for the year of analysis are compared to the coverage that the country would have to have achieved if it were to halve the unserved proportion of its population at a constant rate between 1990 and 2015. A country is considered to be “on-track” if the coverage rate is at least 95% of the requirement, to be “making insufficient progress” if coverage is between 90% and 95% of the required coverage, and “not on track” if coverage is less than 90% of the required coverage. Similar measures are used by the UN for assessing progress for other MDG targets.

Progress is tracked by comparing current to baseline coverage. For the purpose of MDG monitoring, a 1990 baseline is used. However, nationally-representative baseline data from 1990 were not available for all countries at the beginning of the MDG period, and coverage for most countries was interpolated using the same linear regression methods described above. As additional coverage data have become available each year from newly conducted and earlier unrecognised national surveys and censuses [8], and regression lines have been recalculated to fit these, interpolated baseline coverage rates have shifted slightly over time (Table 4). 

**Table 4:** 1990 Baseline Global Coverage Percentages as Estimated in Different Years.

Reporting Year	Use of Improved Drinking Water			Use of Basic Sanitation		
	1990 Baseline		2015 Target	1990 Baseline		2015 Target
	With Access	Without Access		With Access	Without Access	
2000	79	21	11	55	45	23
2004	77	23	12	49	51	26
2006	78	22	11	49	51	26
2008	77	23	12	54	46	23
2010	77	23	12	54	46	23
2012	76	24	12	49	51	26
2013	76	24	12	49	51	26
2014	76	24	12	49	51	26

Increasingly, the JMP has provided selected supplementary analyses in its reports. These have included: wealth quintile analysis (2004, 2008, 2010, and 2012), water collection and household water treatment practices, including the burden of time spent fetching water (2008, 2010, and 2012), disposal of child faeces (2008), and use of bottled water (2010) [35,36,37,84]. In 2011, the JMP issued an in-depth thematic report on drinking water [85]. This included additional analyses of coverage by wealth quintile, household water treatment practices, the use of bottled water, and the sustainability of urban and rural drinking water services, supplementing information from the JMP database with other data. 

## 5. Evolution of the JMP Method 

The JMP periodically consults independent experts on technical and managerial aspects of drinking water and sanitation, monitoring and statistics in order to review and update its methods. JMP has convened task forces on sanitation, methods, water quality, and urban WASH monitoring, and in 2009 formed a standing Scientific Advisory Group. Since 2000, these consultations and task force activities have emphasised: shifting from provider-based to user-based data; ensuring all potential censuses and household surveys are used; standardizing survey questions; assessing drinking water safety; and evaluating alternatives to the linear regression method. The JMP also convened working groups in 2011–2012 to advise on potential future targets and indicators for post-2015 Sustainable Development Goals as well as on the implications of such targets and indicators for monitoring [86].

### 5.1. Change from Provider to User Orientation 

The most substantive change in the overall JMP method was that from provider-based to user-based data in 2000, which has been credited with improving the accuracy and credibility of monitoring [35]. The national agencies that were the sources of data before that time were assumed to be often well-informed about planned development targets and corresponding government implementation efforts, as well as the activities of major utilities; but less well-informed about the proportion of water and sanitation facilities actually functioning or in use, services delivered or managed by local government in smaller towns and cities, spontaneous household or community initiatives, and facilities established with the support of other parties such as non-governmental organisations (NGOs). By contrast, users of facilities were assumed to be aware of the type and functionality of the facilities that they use; and to be willing providers of such information. Well-implemented household surveys were therefore adopted as a means of collecting such user-based information. 

**Figure 3:**
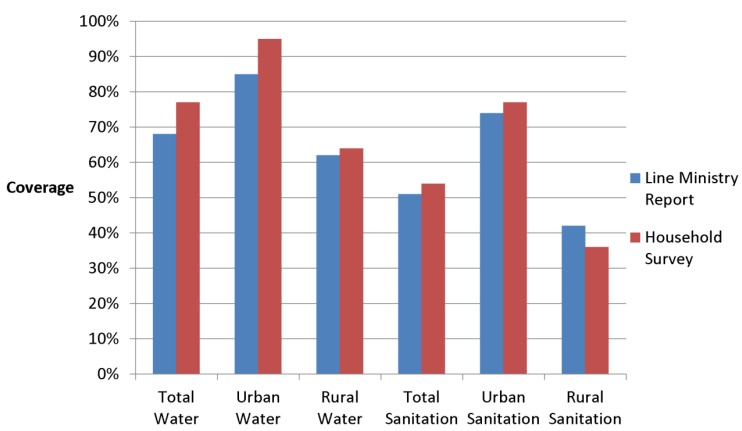
Comparison of 1990 global coverage estimates derived from provider-based line ministry data versus user-based (e.g., household survey and census) data [36,88].

**Figure 4:**
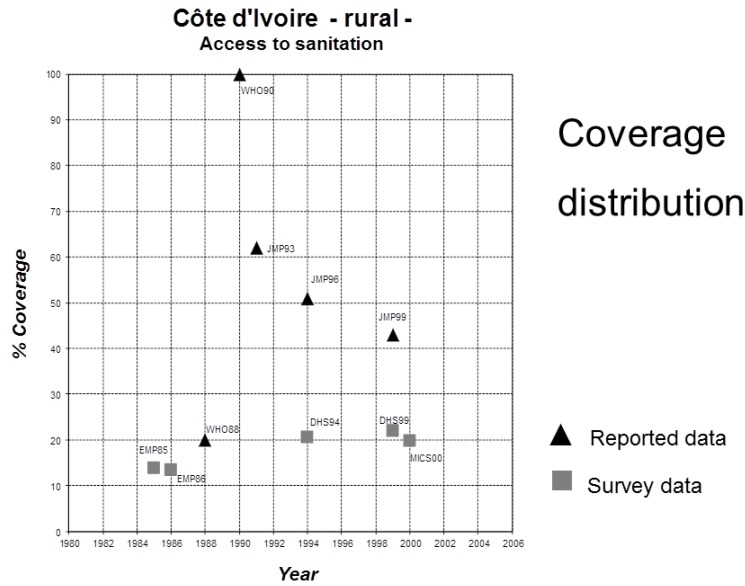
Comparison of line ministry reported rural sanitation coverage rates *vs*. coverage rates calculated from household surveys for Cote d’Ivoire [87].

Data confirm substantive differences in coverage estimates for the same year using the two approaches: 1990 urban drinking water coverage estimates varied by 10 percentage points, while rural sanitation figures varied by 6 percentage points (Table 5, Figure 3); similarly, line ministry sanitation coverage data (for example from Cote d’Ivoire, Figure 4) showed large fluctuations over the 1980–2000 time period, while estimates from household surveys showed gradual growth. While national data and household surveys are often substantially different, the extent and direction of these differences vary greatly across contexts, and thus no blanket correction can be applied. (Figure 3 and Figure 4, Table 5) [87]. 

**Table 5:** Comparison of global coverage estimates derived from line ministry (“provider”) versus household (“user”)—based data, for 1990.

Data Type	Rural		Urban	
	Line Ministry Reporting (1)	Household Survey Based (2)	Line Ministry Reporting (1)	Household Survey Based (2)
Water	68%	62%	85%	95%
Sanitation	51%	28%	74%	76%

Notes: (1): [88]; (2): [8].

In 2014, the JMP database contained data from 1797 data sources. Of these, 252 were censuses, 1211 were household surveys, and 334 were government reports from countries where censuses and surveys no longer include drinking water and sanitation questions. In the period between the 2013 and 2014 reports, 106 new data sources were added: 75 from the period 2008–2012, 19 from 2000–2007 and 12 from the period prior to 2000. With a growing number of data sources, it may be possible to achieve more accurate estimates by weighting surveys and censuses more heavily if they have large sample sizes and/or high quality drinking water and sanitation data (for example, where there is good alignment of response categories with monitoring categories and the “50% rule” described earlier is not applied). In the future, it may also be possible to further expand the JMP database to include data from utilities, regulators, and other providers. However, use of such datasets present challenges, since few are likely to be randomised or nationally representative, and care must be taken to avoid bias.

### 5.2. Standardisation of Survey Questions on Drinking Water and Sanitation

In order to harmonise water- and sanitation-related questions across different household surveys, JMP identified a set of core questions [16] (Table 1), recommended changes to question response categories, and collaborated with the major household surveys (Table 1) to promote the incorporation of these elements. Response category modifications included, for example, descriptive categories for latrines (other than ventilated improved pit latrines and pour-flush latrines): “pit latrine with slab” and “pit latrine without slab”, where a slab is defined as any solid structure that covers the entire pit opening except for the drop hole or seat. The core questions and latrine classifications were included in MICS and DHS surveys from 2005. This change harmonised questions on drinking water and sanitation between these two surveys, and the core questions have not yet been incorporated into all national surveys and censuses.

Further harmonisation of survey questions and classifications is needed for more accurate reporting of coverage and trends by facility class. Such reporting would reduce the noise introduced by correction factors that are currently used in aggregating coverage statistics across countries and surveys with inconsistent facility classifications. In addition, the use of a standard classification system would facilitate continuation of the shift from the “improved/unimproved” dichotomy and towards reporting of information on coverage with defined facility types. Towards this end, the JMP introduced more differentiated reporting (column 3 of Table 1) in 2008 [84]. 

Because household surveys are time-consuming and expensive, there is continuous pressure to reduce the number of questions in order to minimise respondent fatigue and associated loss of data quality; questions must therefore be harmonised to efficiently elicit a large amount of usable and comparable data.

### 5.3. Alternatives to Linear Regression

The JMP has explored alternatives to linear regression that would facilitate more accurate calculation of national and sub-national coverage rates, both at the household level and in extra-household settings such as schools, workplaces, and health care facilities. Proposed alternatives to simple linear regression include linear, nonlinear and cubic spline models, and others that capture departures from linearity in the data [89,90]. The likely impact of using such alternative regression methods is briefly discussed below.

## 6. Discussion

JMP’s monitoring approach has evolved substantially over time, abandoning “sector” information, now reported by GLAAS, and incorporating an increasing amount of survey and census data. The current JMP method has several limitations and biases, both with respect to current applications and potential future monitoring needs. In our discussion, we assess the validity of data produced by current JMP methods and address the need to refine indicators and monitoring methods to inform future international policy and implementation efforts on drinking water and sanitation. These future needs will be further discussed in a forthcoming companion article [12].

### 6.1. Accuracy of Current Methods in Representing the Global Coverage Situation

**Figure 5:**
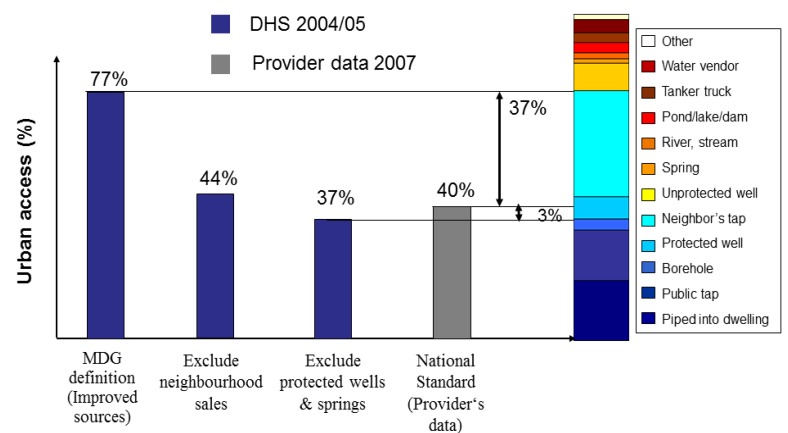
Comparison of line ministry reported drinking water coverage rates *vs.* coverage rates calculated from Household Surveys for Tanzania.

One criticism of JMP coverage estimates has been large discrepancies compared to national estimates. These discrepancies are largely due to two factors: the fact that JMP coverage estimates are derived from linear regressions, rather than the results of the most recent survey in the country; and differences in definitions with respect to the types of facilities that count towards coverage. The 2005 data for drinking water use in urban areas in Tanzania illustrate the latter case. There is a large difference between the JMP-estimated coverage rate of 77% and the national data report of 40%. The JMP and the government of Tanzania differ in how they classify facilities as counting towards coverage. If neighbourhood sales and use of protected springs and wells are included in the national coverage rate, as they are by JMP, then a difference of only 3% remains (Figure 5). While such differences in definitions and service classifications across countries complicate monitoring, they are understandable, since national standards evolve over time as countries progressively achieve higher levels of service, while JMP applies a consistent classification for international compatibility. 

Similar differences are reported between JMP reports and local studies. Measured water quality is a frequent cause of such differences. Furthermore, smaller studies may not be nationally-representative—although they may give valuable insights into local conditions and variability. 

While differences in definitions and representativeness account for some inconsistencies, other differences are likely due to bias and error in JMP estimates, national estimates, the underlying data sources, the JMP method, or a combination of these. 

Errors may be introduced by gaps or biases in the sample frames of nationally representative surveys and the censuses from which they are derived, particularly in countries where certain populations—such as those living in marginal or informal settlements—are excluded from or under-represented in censuses [55]. These groups may also be under-sampled in household surveys that use sample frames from biased or out-of-date national censuses. To the extent that these under-represented disadvantaged groups also experience lower coverage and service levels, this is likely to lead to over-estimation of coverage in the affected countries. Further work is needed to ensure that census sample frames are constructed in a manner that accurately represents all populations and settlements.

Limitations in survey instruments may also result in the introduction of errors and biases. In some cases, survey questionnaire response categories require judgment or interpretation by respondents and/or interviewers as to whether their principal water source is “protected” or not. Where accepted descriptions do not coincide with response options, or where respondents are unable to accurately recall and describe their primary drinking water source, this may lead to misclassification, particularly when sources are not directly observed. Modification of methods to include the direct observation of sources by trained enumerators would help address this.

In addition, household data collection methods can introduce bias. There is the potential for courtesy bias and socially desirable response bias (the tendency for interviewees to give whichever response they believe an interviewer wants to hear, or that the respondent believes would make others view him or her most favourably) in surveys and censuses. For example, if respondents without improved sanitation are predisposed to claim that they regularly use a latrine in order to avoid embarrassment; this could result in over-estimation of sanitation coverage. These effects may be more significant for sanitation than for water. Indeed, it has been suggested that socially desirable response bias may be particularly problematic when assessing the outcome of programs to end open defecation. Such bias can be limited by the use of trained interviewers, properly structured surveys, and the absence of immediate benefit to the respondent [91], but cannot be eliminated. 

Low precision (but not low accuracy) is intrinsic to the survey approach. The use of linear regression addresses this limitation to some extent by using data from multiple surveys, but does not address inaccuracy due to linear modelling of processes that may not be linear in many contexts. It has been suggested that trends in water and sanitation coverage typically follow “S-shaped” curves, rather than linear trends, with a tendency for the rate of progress to slow as countries approach full coverage [89]. If this is the case, linear models likely introduce substantial bias, particularly when coverage rates are near 0% or 100%. Alternatives to linear regression could thus increase accuracy and reduce bias inherent in current methods. The use of weighted models, with greater weights applied to larger surveys and to those of higher quality, could further improve accuracy.

Errors may also be introduced by the rules concerning extrapolation, such as the application of a nominal 50% improved facilities correction where a survey or census does not use facility class definitions that can be mapped to JMP definitions, and interpolation from other surveys for that same country and time period is not possible, as described above. Such errors will tend to most affect data-poor countries, which are more likely to be smaller and less economically developed. The direction of error will depend on each country’s specific circumstances. However, few countries are currently affected by the application of these rules, and their total population is small in global terms.

Bias may also be introduced by calculating and applying a single rate of shared sanitation use for each country across all time periods, instead of periodically updating these figures to reflect changes. 

Bias may also be introduced by JMP’s reliance on household-level data for calculating rural/urban coverage rates. The JMP method involves estimating the proportions of rural and urban *households* using identified facility types and then applying these to the corresponding *populations* to estimate coverage, using average urban and rural household sizes in the calculation. It is plausible that there are associations between household size and the likelihood of having better or worse facilities (for example richer households may tend to be both smaller and have better water and sanitation facilities), which would lead to an over-estimation of population coverage. While this bias could be reduced by basing coverage estimates on individual household population data, this would be time-consuming and would not be possible where “raw” data are unavailable and JMP relies on survey reports issued by the office carrying out the survey. 

Analyses are further complicated by the lack of a common definition of rural and urban. Under current definitions, rural/urban boundaries are often arbitrary, and peri-urban areas can be difficult to classify. Such definitions have the potential to significantly impact reported differences in coverage rates when stratified by this variable [92]. Furthermore, intra-urban disparities are rarely captured. Recent work has suggested several geospatial methods for assessing “urban-ness”, and these may prove useful in studying rural/urban inequalities across different contexts, as well as in considering more nuanced approaches to disaggregation by “urban-ness” (idem).

In both surveys and censuses, it is implicitly assumed that households use a single source for all their domestic water needs, and that the situation at the time of survey is annually representative. This assumption is problematic. In many settings households use a “portfolio” of water sources, varying by time of year, type of use, and functionality of each source. Furthermore, water source quality and reliability may also vary by season [93]. Similarly, both children and adults may use different sanitation facilities when at home and when away from the home (especially at work or school); and the concept of a “household” can vary widely between cultures. The direction of errors introduced by focusing on a single set of household-level facilities for estimating drinking water and sanitation coverage will depend on specific circumstances and may lead to over- or under-estimation of coverage. Seasonal and functionality effects are more likely to impact drinking water than sanitation estimates using current methods.

The generalisations implicit in facility classification (Table 1) lead to some households being categorised as unserved despite receiving adequate services. For example, water delivered by tanker trucks may in fact serve as a source of sufficient safe water in some circumstances. This is reflected in the classification of such services as “improved” in the United Arab Emirates, Oman and Kuwait, where this is a regulated service provided by the drinking water authorities to nomadic populations and communities not connected to a piped network. Similarly, in some circumstances sanitation services involving cartage (transport of excreta in containers such as buckets) are managed hygienically. There has been extensive discussion on the acceptability of shared sanitation facilities if used by a limited number of households whose members know each other [94]. Changes in the definitions of these categories would have large impacts. For example, if a measure of the quality of the water were incorporated into the definition of an improved drinking water facility, the drinking water component of the MDG target would be badly off track [95]; and if households sharing sanitation facilities otherwise classified as “improved” were considered to have adequate sanitation, then the sanitation component of the MDG target would be judged to be on track. Finally, while travel time to source has been recently considered [36], the distance component of access (as defined by JMP) is not currently factored into estimates, leading to coverage overestimation of unknown magnitude [96]. Affordability has also not been addressed (idem).

### 6.2. Future Monitoring Needs

Monitoring often involves simplification in order to describe complex realities with a few simple, indicators that are adequate to inform policy and practice. Over many decades, the principal simplification in international monitoring of drinking water and sanitation has been to reduce each to a single parameter (use of an improved facility type), assessed at household level, applied to all household members, and expressed as coverage at the national level for rural and urban populations. However, the high and increasing levels of coverage so assessed and the growing recognition of the human right to water and sanitation has led to an increased demand for disaggregated monitoring that can facilitate comparison of access levels across demographic and ethnic sub-groups, as well as by gender. Improved geospatial disaggregation of monitoring data could also help reveal hidden inequalities [97]. Furthermore, simple linear regressions do not capture progressive realisation of the human right to water and sanitation, although recently developed methods may be able to do so using JMP data [98]. The use of alternative methods (addressed above) to analyse increasingly disaggregated data may thus be better able to support reporting on progressive realisation, in addition to improving the accuracy of coverage estimates.

While the MDG-period’s simple binary approach of reporting on “the haves and the have-nots” [11] has made the JMP findings easily absorbable by a broad audience, this approach has substantive limitations, stemming from the fact that different facility types are associated with different types and levels of benefits. While the refinement of JMP reporting beyond the improved/unimproved dichotomy (discussed in Section 5.3) begins to address these limitations, further disaggregation by class could inform the use of service ladders to rank facility classes by their desirability from a health perspective. 

Furthermore, the service quality and benefits of individual facilities can also vary widely. Many improved sources are contaminated with faecal bacteria, while some unimproved sources may produce water that is free from such contamination [99]. Chemical contamination of improved sources is likewise a serious concern [96]. There is growing interest in coverage definitions that incorporate water quality and accessibility, relative to minimum benchmarks for each. Water quality testing has been included in household surveys on a pilot basis [100], and JMP is exploring ways to incorporate water quality indicators into post-2015 monitoring. Further monitoring could facilitate disaggregation and/or correction of coverage statistics to account for microbial water quality variables such as faecal bacteria concentration, as well as drinking water safety indicators such as sanitary inspection scores [101]. Furthermore, as additional chemical water quality data become available, corrections such as those made at the country level for arsenic in Bangladesh may become feasible and useful at both national and sub-national scales elsewhere. As monitoring efforts expand to generate increasing amounts of water safety information, the JMP method must evolve to allow the incorporation and stratified analyses of these data. 

There is increasing recognition of the need for a more comprehensive “service quality” or “service ladder” approach that accounts for the different levels of service provided by various drinking water and sanitation facilities, and their associated benefits [8,102]. Since most surveys distinguish households where water is piped into the dwelling from those in which it is available on plot or in the yard, coverage could be disaggregated in this way. Water source functionality and reliability impact the level of service provided by sources within a given facility class, and could be included in future analyses as well. For households without access to safe, continuous household-level piped supplies, some measure of the safety of household drinking water storage methods may also be of use. In the case of sanitation, it is helpful to distinguish between unimproved facilities based on their effectiveness at separating users and the wider population from human excreta [8]. Further work is needed to develop and standardise operational definitions of key service quality metrics, and incorporate these into stratified analyses and coverage estimates.

While the focus on households as the primary concern is logical, securing the benefits of drinking water and sanitation requires that facilities be readily available to users in all settings, including schools, workplaces, hospitals and clinics, as well as refugee camps. The JMP has considered options for targets and indicators for the post-2015 period that would address these concerns [86,103]. 

While measuring water and sanitation coverage is the primary objective of JMP monitoring, improved metrics for and additional data on the burden of fetching water, the availability of water for productive uses, and the prevalence of open defecation-free communities are examples of additional indicators that might be of interest to policy-makers, researchers, and implementers. 

The data needed to inform policy and practice are different at global, national, and sub-national levels. For example, national stakeholders may need more disaggregated data, while for global purposes it may be enough to know how many people are accessing different kinds of services in rural and urban settings. For local management purposes, it is important to know more about the functionality of individual schemes, and also about regional distributions. There is scope for greater harmonisation of monitoring and reporting across these levels to increase efficiency so that local and national stakeholders can make better use of global monitoring data, and vice versa.

## 7. Conclusions 

Since the initiation of international drinking water and sanitation monitoring under the UN system, methods have evolved significantly, and the volume and quality of available data has increased rapidly. The internationally comparable data collected by JMP for global drinking water and sanitation coverage over the MDG period, as well as the resulting estimates, have been of considerable use to stakeholders and policy makers. However, several studies have identified limitations in the current method, as well as opportunities for improvement. Integrating these improvements into international monitoring is a long-term commitment that requires confidence in the proposed indicators and data collection methods, as well as in their medium- to long-term relevance. 

Since 2000, JMP monitoring has been facilitated by the availability of large amounts of data from national censuses and household surveys at no direct cost. Using these data, JMP can calculate coverage with improved and unimproved drinking water and sanitation facilities, as well as piped water and shared sanitation, at the national, regional, and global levels. However, current data and resource limitations make it challenging for JMP to estimate coverage for individual facility classes, address water safety issues, or address equity in its estimates. Some of the future options under discussion, such as addressing water safety, require data that are not currently collected in available surveys and censuses. In some cases, existing data may be available from regulators and other national entities, while in other cases dedicated data collection would be required, with associated costs. 

There is a tension between systematic monitoring sustained over decades and the demand for new targets and indicators with short intervals that match political cycles. While it is commonly perceived that policy drives and monitoring follows, the MDG experience makes clear that, in practice, effective monitoring must anticipate policy needs. It is noteworthy that it required roughly a decade to validate the JMP’s current methods for monitoring drinking water and sanitation at the global scale, establish basic capacities, and collect baseline data world-wide. 

As today, effective monitoring will continue to require identification of indicators and collection of data that enable reliable comparisons between countries and over time. Effective *international* monitoring will also require central compilation and quality assurance to achieve those ends. 

It is likely that drinking water and sanitation targets and indicators for the 2015–2040 period will include more refined measures of water safety, equity, and service level, and will feature greater disaggregation across demographic and geographic dimensions. Improved analytical methods may also facilitate more accurate estimates of coverage and service quality at the global, national, and sub-national levels. Finally, future methods may consider the expansion of data sources and collection systems to include information from regulators, utilities, non-governmental organizations, and perhaps eventually even individual users, if practicable.

As countries move towards higher rates of coverage, improved monitoring indicators and methods will prove useful in tracking the efficiency and equity with which remaining unserved populations are reached, as well as the rate at which populations with basic service gain access to higher levels of drinking water and sanitation services.
